# Resting‐State EEG Aperiodic Activity and Negative Affective Vulnerability: The Mediating Role of Big Five Personality Traits

**DOI:** 10.1002/brb3.71836

**Published:** 2026-10-21

**Authors:** Jin Lin, Zhiqing Dai, Sitong Feng, Ruiwen Song, Xinyue Jiang, Huanshu Xu, Ziyi Zhao, Hongpeng Lv, Hongxiao Jia

**Affiliations:** ^1^ Beijing Key Laboratory of Intelligent Drug Research and Development for Mental Disorders, National Clinical Research Center for Mental Disorders, National Center For Mental Disorders, Beijing Anding Hospital Capital Medical University Beijing China; ^2^ School of Management Tianjin University of Traditional Chinese Medicine Tianjin China; ^3^ Beijing University of Chinese Medicine Third Affiliated Hospital Beijing China

**Keywords:** aperiodic exponent, Big Five personality traits, resting‐state EEG, trait anxiety

## Abstract

**Introduction:**

Resting‐state EEG aperiodic parameters characterize the broadband background component of neural activity. The offset reflects overall broadband power and has been linked to population‐level firing, whereas the exponent is often interpreted, within a theoretical framework, as an indirect proxy for cortical excitation/inhibition (E/I) balance. These measures may therefore provide candidate neurophysiological trait markers of negative affective vulnerability, but it remains unclear whether their associations with trait anxiety and trait depression are direct or are statistically accounted for by stable personality traits.

**Methods:**

A total of 164 healthy adults completed measures of the Big Five personality traits, trait anxiety, and trait depression and underwent eyes‐closed resting‐state EEG recording. Aperiodic exponent and offset were estimated at the whole‐brain and regional levels using spectral parameterization. Partial correlations, hierarchical regression analyses, and bootstrap mediation analyses were conducted controlling for sex, age, and years of education.

**Results:**

Neuroticism showed the strongest and most spatially widespread positive associations with the aperiodic exponent. Trait anxiety showed more limited associations, mainly with whole‐brain and frontal aperiodic parameters. The association between whole‐brain exponent and trait anxiety was no longer significant after the Big Five personality traits were entered into the model. Bootstrap mediation analyses identified significant indirect effects in this association through personality traits, with neuroticism showing the largest indirect effect and conscientiousness also showing a significant indirect effect. Trait depression was not directly associated with aperiodic parameters, although significant indirect effects through personality traits were observed.

**Conclusions:**

Resting‐state EEG aperiodic activity, particularly the exponent, was more consistently associated with stable personality dispositions than with negative affective vulnerability directly. Personality traits, especially neuroticism, may statistically account for part of the association between aperiodic exponent and trait anxiety. Because the data were cross‐sectional, the temporal and causal direction of these associations cannot be determined.

## Graphical abstract

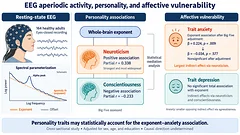

**Abbreviations**
CI: Confidence interval
E/I: Excitation/inhibition
EEG: Electroencephalography
FDR: False discovery rate
FOOOF: Fitting Oscillations and One‐Over‐F
GABA: Gamma‐aminobutyric acid
GAD1: Glutamate decarboxylase 1
ICA: Independent component analysis
MRS: Magnetic resonance spectroscopy
NEO‐FFI: Neuroticism Extraversion Openness Five‐Factor Inventory
NEO‐PI‐R: Revised NEO Personality Inventory
SD: Standard deviation
STAI‐T: Trait subscale of the State–Trait Anxiety Inventory
STDI‐T: Trait subscale of the State–Trait Depression Inventory.

## 1 Introduction

Personality traits reflect relatively enduring patterns of emotion and behavior, thereby offering a framework for understanding why individuals vary in their susceptibility to anxiety, depression, and psychological distress when confronted with comparable stressors (Kotov et al. 2010; Yang et al. 2023). Negative affective vulnerability is commonly conceptualized as an important dimension of individual differences, reflecting a stable tendency to experience negative emotions such as anxiety, worry, and sadness (Watson and Clark 1984). Trait anxiety and trait depression constitute two core manifestations of this dimension, reflecting stable tendencies to experience anxiety‐ and depression‐related emotions over time. Both are widely regarded as important vulnerability factors for a spectrum of emotional disorders (Knowles and Olatunji 2020; Song et al. 2023).

With the high temporal resolution, resting‐state electroencephalography (EEG) serves as an important tool for investigating the neural basis of individual differences (Lopez et al. 2023). Prior research has predominantly focused on periodic oscillatory activity, such as alpha‐ and beta‐band rhythms, whereas emerging evidence indicates that the aperiodic component of the power spectrum is not simply background noise but also carries physiologically meaningful information (Donoghue et al. 2020). The aperiodic component of the EEG power spectrum can be decomposed into two indices: the offset and the exponent. The exponent, in particular, has been theorized to serve as a proxy for cortical excitation/inhibition (E/I) balance. Cortical E/I characteristics exhibit substantial interindividual variability among healthy populations, with such heterogeneity hypothesized to underlie stable individual differences in personality and affective traits (Bolden et al. 2017; Wu et al. 2025). Collectively, these findings suggest that cortical dynamic properties indexed by the aperiodic component may be intrinsically related to individual differences in negative affective vulnerability and personality traits, thereby providing a neurophysiological perspective on individual differences in personality and affective characteristics.

Despite these theoretical advances, direct evidence linking EEG aperiodic components to personality and negative affective vulnerability remains scarce and inconsistent. On the one hand, existing studies have yet to reach a clear consensus regarding which specific dimensions are involved and whether the observed associations are unidirectional or bidirectional (Fruehlinger et al. 2024; Pacheco et al. 2024). On the other hand, existing studies have tended to examine either personality or affective variables in isolation, with relatively few addressing the hierarchical relationships among personality traits, negative affective vulnerability, and aperiodic neural activity within a single integrative framework. Critically, it remains unclear whether the associations between aperiodic components and trait anxiety and trait depression are direct or are instead primarily expressed indirectly through stable personality dispositions. Moreover, whether offset and exponent differ in their sensitivity to personality and negative affective vulnerability also warrants further investigation.

The present study aimed to characterize the associations among resting‐state EEG aperiodic parameters, Big Five personality dimensions, and negative affective vulnerability in a healthy adult cohort. Mediation analyses were further conducted to examine whether the associations between aperiodic parameters and negative affective vulnerability were statistically accounted for by the Big Five personality traits. We hypothesized that aperiodic components would exhibit direct associations with personality traits, while their associations with negative affective vulnerability would be primarily expressed indirectly through personality traits. Integrating neurophysiological, personality, and affective measures, this study provides a multilevel perspective on the neurophysiological correlates of individual differences in negative affective vulnerability.

## 2 Materials and Methods

### 2.1 Participants

Participants were recruited from April 2025 to February 2026 through on‐site advertisements at Beijing Anding Hospital, Capital Medical University, and surrounding communities, as well as through campus advertisements and online recruitment notices. All psychological assessments and EEG recordings were conducted in a controlled laboratory environment at Beijing Anding Hospital, Capital Medical University.

The inclusion criteria were as follows: (1) age 18‐55 years; (2) right‐handedness; (3) capacity to understand and independently complete the psychological assessments and resting‐state EEG recording; (4) normal or corrected‐to‐normal visual acuity; and (5) provision of written informed consent.

The exclusion criteria were as follows: (1) current or lifetime diagnosis of psychiatric or neurological disorder; (2) history of traumatic brain injury, epilepsy, loss of consciousness, or any major medical conditions with potential central nervous system impact; (3) current psychotropic or other medication use with substantial central nervous system function; (4) history of alcohol or substance abuse disorder; (5) incomplete or invalid questionnaire responses; (6) failure to complete the study procedures or withdrawal from the study; and (7) insufficient EEG data quality.

To minimize short‐term lifestyle confounds on EEG recordings, all participants were instructed to avoid sleep deprivation prior to EEG recording, abstain from alcohol and psychoactive substances within 24 h before testing, and refrain from caffeinated beverages within 2 h before testing.

The initial sample comprised 171 participants. Seven participants were excluded due to poor EEG data quality. The final sample included 164 participants, of whom 110 were female (67.1%), with a mean age of 20.08 ± 2.00 years and a mean education duration of 14.13 ± 1.70 years.

The study was approved by the Ethics Committee of Beijing Anding Hospital, Capital Medical University (approval no. (2025) Research Ethics Approval No. 123). All participants provided written informed consent prior to their participation. The study was conducted in accordance with the Declaration of Helsinki.

## 3 Questionnaires

### 3.1 Trait Anxiety

Trait anxiety was assessed using the Trait subscale of the State–Trait Anxiety Inventory (STAI‐T) (Spielberger et al. 1983). The scale consists of 20 items rated on a 4‐point Likert scale ranging from 1 (almost never) to 4 (almost always), with total scores ranging from 20 to 80. Higher scores reflect higher levels of trait anxiety.

### 3.2 Trait Depression

Trait depression was assessed using the Trait subscale of the State–Trait Depression Inventory (STDI‐T) (Spielberger et al. 2005). The scale consists of 16 items rated on a 4‐point scale, with total scores ranging from 16 to 64. Higher scores indicate higher levels of trait depression.

### 3.3 Personality Traits

Personality traits were measured using the Chinese version of the Neuroticism Extraversion Openness Five‐Factor Inventory (NEO‐FFI) (Yang and Li 2010), a 60‐item condensed version of the NEO Personality Inventory‐Revised (NEO‐PI‐R) (Costa and McCrae 1992). It comprises five dimensions—neuroticism, extraversion, openness, agreeableness, and conscientiousness—with 12 items for each dimension, yielding a total of 60 items. All items are rated on a 5‐point Likert scale ranging from 1 (strongly disagree) to 5 (strongly agree). Mean scores for each dimension (range: 1–5) were used as analytic variables.

### 3.4 EEG Recording and Pre‐Processing

EEG data were recorded using a 64‐channel acquisition system (Brain Products, BrainAmp MR 32). Ag/AgCl electrodes were mounted in an elastic cap (GREENTEK, Wuhan, China) and positioned according to the international 10–20 system. Signals were sampled at 1000 Hz with electrode impedances maintained below 10 kΩ using conductive gel (GT5 conductive gel, GREENTEK, Wuhan, China). During acquisition, participants were seated quietly in a sound‐attenuated, temperature‐ and humidity‐controlled shielded room and underwent eyes‐closed resting‐state EEG recording. After signal stabilization, at least 5 min of eyes‐closed resting‐state EEG data were collected using BrainVision Recorder (Version 2.0, Brain Products).

EEG pre‐processing was conducted in MATLAB (Version R2024b, MathWorks, USA) and the EEGLAB toolbox. Standard channel location information was first imported. Continuous EEG data were then band‐pass filtered at 0.5‐45 Hz and notch filtered at 48‐52 Hz to reduce low‐frequency drift, high‐frequency noise, and line noise contamination. The data were subsequently downsampled to 500 Hz, re‐referenced offline to the average of all scalp electrodes (online reference: FCz), and further processed by rejecting artifact‐contaminated segments, interpolating bad channels, and performing independent component analysis (ICA). Components corresponding to ocular, muscular, and movement artifacts were identified and removed through visual inspection, yielding clean EEG signals for subsequent analysis.

Based on the spatial distribution of electrodes in the international 10‐20 system, the 61 EEG electrodes were further grouped into six regions of interest according to their anatomical locations: prefrontal, frontal, central, parietal, temporal, and occipital regions. The electrodes included in each region were as follows: prefrontal region (Fp1, Fp2, Fpz, AF3, AF4, AF7, AF8), frontal region (F1, F2, F3, F4, F5, F6, F7, F8, Fz), central region (C1, C2, C3, C4, C5, C6, Cz, FC1, FC2, FC3, FC4, FC5, FC6), parietal region (P1, P2, P3, P4, P5, P6, P7, P8, Pz, CP1, CP2, CP3, CP4, CP5, CP6, CPz), temporal region (T7, T8, TP7, TP8, TP9, TP10, FT7, FT8), and occipital region (O1, O2, Oz, PO3, PO4, PO7, PO8, POz).

### 3.5 Fitting the 1/f Slope

Prior to FOOOF fitting, power spectral density was estimated separately for each channel using Welch's method with 4‐s Hamming‐windowed segments and 50% overlap between adjacent segments. The FFT length was set equal to the window length, yielding a frequency resolution of 0.25 Hz. The 4‐s window was selected to balance frequency resolution and the number of segments available for averaging, while the 50% overlap was used to improve the stability of the spectral estimates. Aperiodic 1/f characteristics were parameterized using the “FOOOF” algorithm: Fitting oscillations and 1/f’ package in Brainstorm (Donoghue et al. 2020). The frequency range for analysis was set at 1‐40 Hz with a Gaussian peak model (peak width limits = 0.5‐12 Hz, maximum number of peaks = 3, minimum peak height = 3 dB, proximity threshold = 2 standard deviations (SD) of the peak model, and a fixed aperiodic model). This approach generated participant‐specific estimates of aperiodic exponent and offset. These settings were selected in accordance with established FOOOF recommendations and the Brainstorm implementation. The constraints on peak width, number, height, and proximity were applied to reduce the fitting of noise‐related, overlapping, or boundary‐adjacent peaks and thereby limit overfitting.

### 3.6 Statistical Analysis

All statistical analyses were conducted in R version 4.3.1. Descriptive statistics were calculated for all study variables, and internal consistency was evaluated using McDonald's ω. Pearson correlations were used to examine associations among psychological variables.

Partial correlations were computed between whole‐brain and regional aperiodic parameters (exponent and offset) and the psychological variables, controlling for sex, age, and years of education. P values were adjusted using the Benjamini–Hochberg false discovery rate (FDR) procedure, with *q* < 0.05 considered statistically significant. Hierarchical linear regression analyses were performed to examine the incremental predictive utility of whole‐brain aperiodic parameters for psychological variables. Sex, age, and years of education were entered in Step 1. The whole‐brain exponent or offset was entered in Step 2 in parallel models. For trait anxiety and trait depression, the five Big Five personality dimensions were additionally entered in Step 3.

Bootstrap mediation analyses with 10,000 resamples were conducted to evaluate whether personality traits mediated the associations between whole‐brain aperiodic parameters and trait anxiety or trait depression, controlling for sex, age, and years of education. First, simple mediation models were tested with neuroticism as the sole mediator. Second, parallel multiple mediation models were examined with all the Big Five personality traits entered simultaneously as mediators. The significance of indirect effects was determined by a 95% bias‐corrected and accelerated confidence interval that did not include zero. Regional specificity was assessed via simple mediation with regional exponents and neuroticism. Because all variables were assessed cross‐sectionally, these analyses do not establish temporal precedence or causal mediation.

## 4 Results

### 4.1 Descriptive Statistics and Preliminary Analyses of Trait Measures

A total of 164 participants were included in the final analysis, including 110 females (67.1%). All psychological measures showed good internal consistency, with McDonald's ω ranging from 0.872 to 0.916. The VIFs for the Big Five personality dimensions ranged from 1.14 to 1.69, indicating no evidence of problematic multicollinearity. Descriptive statistics and Pearson intercorrelations were presented in Table 1. Trait anxiety was strongly positively correlated with trait depression (*r* = 0.774, *p* < 0.001). Among the Big Five dimensions, neuroticism showed the strongest positive associations with both trait anxiety (*r* = 0.679, *p* < 0.001) and trait depression (r = 0.595, *p* < 0.001), whereas extraversion was negatively correlated with trait depression (*r* = −0.604, *p* < 0.001) and conscientiousness was negatively correlated with trait anxiety (*r* = −0.587, *p* < 0.001).

**TABLE 1:** Descriptive statistics, internal consistency, and intercorrelations among psychological variables (*N* = 164).

Variable	*M*	*SD*	*ω*	*VIF*	1	2	3	4	5	6	7
1. Neuroticism	2.97	0.61	0.882	1.69	—						
2. Extraversion	3.10	0.58	0.886	1.36	−0.461***	—					
3. Openness	3.51	0.52	0.874	1.14	−0.224**	0.241**	—				
4. Agreeableness	3.17	0.41	.872	1.25	−0.325***	0.173*	0.144	—			
5. Conscientiousness	3.48	0.53	.897	1.40	−0.474***	0.330***	0.202**	0.319***	—		
6. Trait Anxiety	42.99	8.87	.892	—	0.679***	−0.482***	−0.156*	−0.188*	−0.587***	—	
7. Trait Depression	30.70	8.26	.916	—	0.595***	−0.604***	−0.213**	−0.221**	−0.448***	0.774***	—

*Note*: ω = McDonald's omega. *** *p* < 0.001, ** *p* < 0.01, and * *p* < 0.05

### 4.2 Partial Correlations Between EEG Aperiodic Components and Psychological Variables

To control for the potential confounding effects of demographic variables, partial correlation analyses were conducted to examine the associations between aperiodic parameters and psychological variables, controlling for sex, age, and years of education. Results were presented in Table 2 and Figure 1. After FDR correction, trait anxiety was positively correlated with the whole‐brain and frontal exponent, as well as with the prefrontal, frontal, and temporal offset, whereas trait depression showed no significant associations with any aperiodic parameter.

**TABLE 2:** Partial correlations between aperiodic parameters and psychological variables (*N* = 164).

Variable	Whole‐brain	Prefrontal	Frontal	Central	Parietal	Temporal	Occipital
**A. Aperiodic Offset**							
Trait Anxiety	0.196	0.201*	0.232**	0.165	0.134	0.237**	0.157
Trait Depression	0.075	0.073	0.134	0.059	0.023	0.118	0.034
Neuroticism	0.238**	0.302***	0.269***	0.213**	0.153	0.283***	0.106
Extraversion	−0.058	−0.071	−0.081	−0.031	−0.021	−0.108	−0.035
Openness	−0.062	−0.119	−0.056	−0.041	−0.013	−0.105	−0.085
Agreeableness	−0.093	−0.151	−0.136	−0.065	−0.026	−0.150	−0.045
Conscientiousness	−0.167	−0.218**	−0.166	−0.131	−0.135	−0.139	−0.161
**B. Aperiodic Exponent**							
Trait Anxiety	0.205**	0.169	0.211**	0.177	0.165	0.190	0.126
Trait Depression	0.112	0.083	0.150	0.106	0.078	0.105	0.058
Neuroticism	0.338***	0.336***	0.326***	0.310***	0.257***	0.317***	0.109
Extraversion	−0.121	−0.127	−0.178	−0.139	−0.029	−0.126	−0.016
Openness	−0.174	−0.119	−0.143	−0.139	−0.137	−0.176	−0.182
Agreeableness	−0.203**	−0.192	−0.186	−0.186	−0.121	−0.256**	−0.100
Conscientiousness	−0.233**	−0.201*	−0.193	−0.161	−0.238**	−0.161	−0.210**

*Note*. Partial correlations controlling for sex, age, and years of education. Significance was determined using Benjamini–Hochberg false discovery rate (FDR) correction across 98 comparisons (*q* < 0.05). Only values surviving FDR correction are marked with asterisks.

***q < 0.001, **q < 0.01, and *q < 0.05.

**FIGURE 1:**
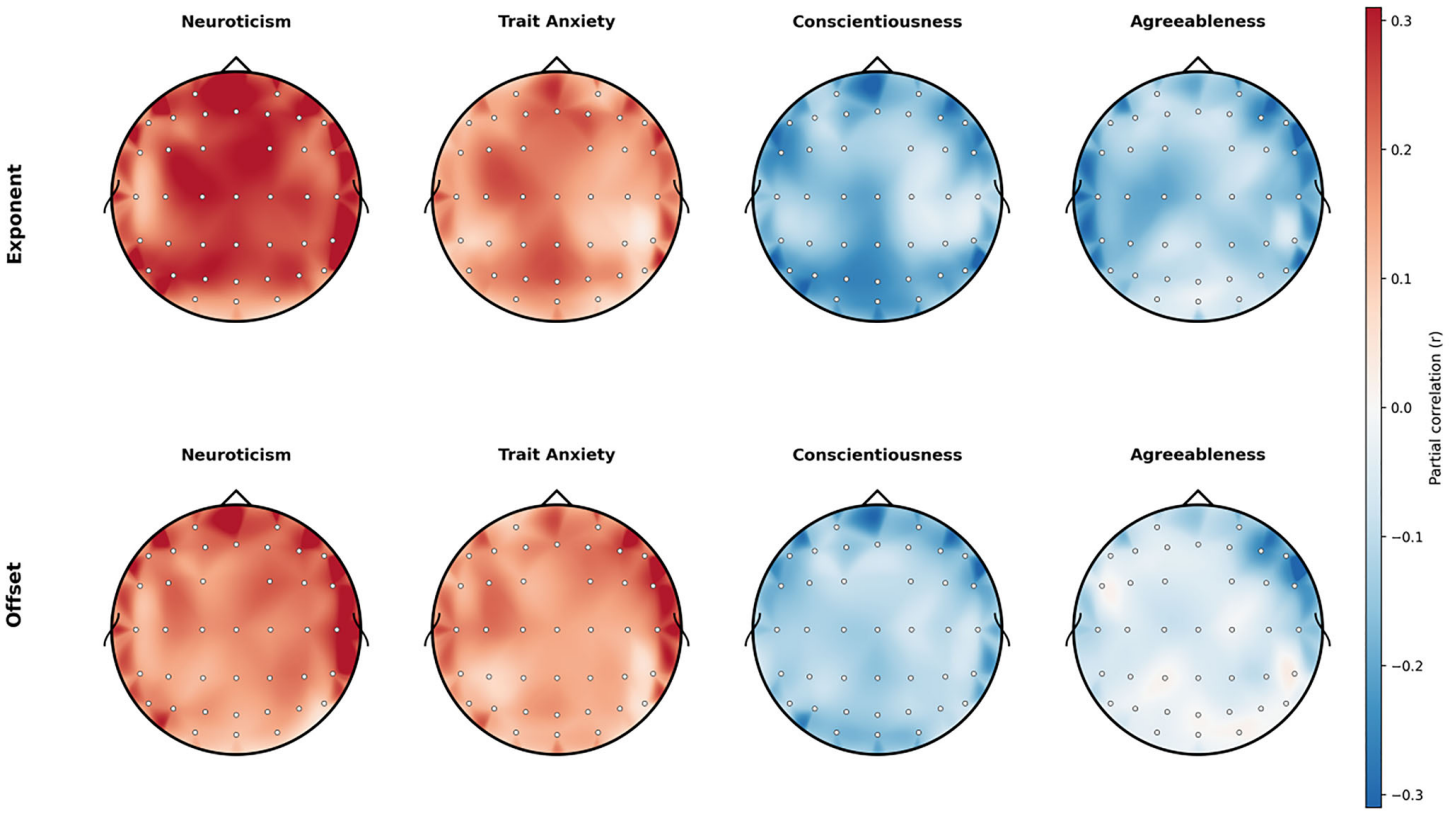
Topographic partial correlations. Topographic distributions of unthresholded channel‐level partial correlation coefficients, controlling for sex, age, and years of education, between aperiodic exponent/offset and psychological variables. Red indicates positive correlations, and blue indicates negative correlations. The maps are presented to illustrate the spatial distribution of effect sizes. Statistical significance was evaluated for the whole‐brain and region‐averaged analyses using Benjamini–Hochberg FDR correction across 98 comparisons (*q* < 0.05).

Among the Big Five personality dimensions, neuroticism showed the most extensive associations with aperiodic parameters. Specifically, neuroticism was positively correlated with exponent across the whole‐brain, prefrontal, frontal, central, parietal, and temporal regions, and with offset across the whole‐brain, prefrontal, frontal, central, and temporal regions. Conscientiousness was negatively correlated with the exponent in the whole‐brain, prefrontal, parietal, and occipital regions, and with prefrontal offset. Agreeableness was negatively correlated with the whole‐brain and temporal exponent, whereas extraversion and openness showed no significant associations with any aperiodic parameter.

### 4.3 Hierarchical Regression Analyses

#### 4.3.1 Associations of Whole‐Brain Aperiodic Parameters With Psychological Variables

Hierarchical regression analyses were conducted to examine the incremental predictive value of whole‐brain aperiodic parameters for psychological variables (Table 3). After adjustment for sex, age, and years of education, whole‐brain exponent most strongly predicted neuroticism (Δ*R* ^2^ = 0.114, *F*‐change = 20.533, *p* < 0.001, β = .369), accounting for an additional 11.4% of the variance. Smaller but significant effects were also observed for conscientiousness, trait anxiety, agreeableness, and openness. Yet, extraversion and trait depression were not significantly predicted.

**TABLE 3:** Hierarchical regression predicting psychological variables (*N* = 164).

Dependent variable	*R* ^2^ Step 1	*R* ^2^ Step 2	Δ*R* ^2^	*F*‐change	*B* (SE)	β	*p*
**A. Whole‐Brain Aperiodic Exponent**							
Neuroticism	0.006	0.120	0.114	20.533***	1.351 (0.298)	**0.369**	**< 0.001**
Conscientiousness	0.007	0.061	0.054	9.146**	−0.807 (0.267)	**−0.255**	**0.003**
Trait Anxiety	0.002	0.044	0.042	6.949**	11.917 (4.521)	**0.224**	**0.009**
Agreeableness	0.055	0.094	0.039	6.826**	−0.531 (0.203)	**−0.216**	**0.010**
Openness	0.034	0.064	0.029	4.984*	−0.591 (0.265)	**−0.188**	**0.027**
Extraversion	0.014	0.028	0.015	2.366	−0.457 (0.297)	−0.132	0.126
Trait Depression	0.002	0.015	0.013	2.031	6.089 (4.273)	0.123	0.156
**B. Whole‐Brain Aperiodic Offset**							
Neuroticism	0.006	0.062	0.056	9.531**	0.641 (0.208)	**0.238**	**0.002**
Trait Anxiety	0.002	0.041	0.038	6.323*	7.682 (3.055)	**0.196**	**0.013**
Conscientiousness	0.007	0.035	0.028	4.563*	−0.390 (0.182)	**−0.167**	**0.034**
Agreeableness	0.055	0.064	0.008	1.392	−0.165 (0.140)	−0.091	0.240
Trait Depression	0.002	0.008	0.006	0.899	2.742 (2.892)	0.075	0.345
Openness	0.034	0.038	0.004	0.612	−0.142 (0.181)	−0.061	0.435
Extraversion	0.014	0.017	0.003	0.530	−0.147 (0.201)	−0.057	0.468

*Note*: Step 1 = sex, age, years of education. Step 2 = + whole‐brain aperiodic parameter. β = standardized coefficient of aperiodic parameter in Step 2. Results within each panel ordered by Δ*R* ^2^. ***p < 0.001, **p < 0.01, and *p < 0.05.

Parallel analyses using whole‐brain offset revealed a similar but attenuated pattern. Offset significantly predicted neuroticism, trait anxiety, and conscientiousness but exhibited no significant associations with agreeableness, openness, extraversion, or trait depression.

#### 4.3.2 Big Five Personality Dimensions as an Explanation for the Association Between Whole‐Brain Exponent and Negative Affective Vulnerability

As shown in Table 4, the association between whole‐brain exponent and trait anxiety was no longer significant after the Big Five personality traits entered into the model. The whole‐brain exponent accounted for significant incremental variance in trait anxiety at Step 2 (Δ*R* ^2^ = 0.042, *F*‐change = 6.949, *p* = 0.009; β = 0.224), but this association was no longer significant after the Big Five traits were added (β = −0.034, *p* = 0.577). The final model explained 59.2% of the variance in trait anxiety, with neuroticism (β = 0.487, *p* < 0.001) and conscientiousness (β = −0.348, *p* < 0.001) emerging as the strongest predictors. Extraversion (β = −0.188, *p* = 0.002) and agreeableness (β = 0.119, *p* = 0.040) were also significant. A similar pattern was observed for whole‐brain offset. Although offset significantly predicted trait anxiety at Step 2 (β = 0.196, *p* = 0.013), this association was no longer significant after the Big Five traits were included in the model (β = 0.030, *p* = 0.576). By contrast, the whole‐brain exponent did not significantly predict trait depression at Step 2 (Δ*R* ^2^ = 0.013, β = 0.123, *p* = 0.156). However, the inclusion of the Big Five traits substantially improved model fit (Δ*R* ^2^ = 0.508, *F*‐change = 32.796, *p* < 0.001), and the final model explained 52.3% of the variance in trait depression. In this model, extraversion (β = −0.395, *p* < 0.001) and neuroticism (β = 0.368, *p* < 0.001) were the strongest predictors.

**TABLE 4:** Hierarchical regression predicting anxiety and depression from whole‐brain aperiodic exponent (*N* = 164).

Step Variable	*R* ^2^	Δ*R* ^2^	*F*‐change	*B* (SE)	β	*p*
**A. Predicting Trait Anxiety (Whole‐brain Exponent)**						
**Step 1: Demographics**	0.002	0.002	0.131			0.941
**Step 2: + Exponent**	0.044	0.042	6.949**			**0.009**
Sex				1.194 (1.587)	0.063	0.453
Age				0.167 (0.626)	0.038	0.790
Education				−0.332 (0.731)	−0.064	0.650
Whole‐brain exponent				11.917 (4.521)	**0.224**	**0.009**
**Step 3: + Big Five**	0.592	0.548	41.374***			**< 0.001**
Sex				−1.418 (1.088)	−0.075	0.194
Age				−0.355 (0.427)	−0.080	0.407
Education				0.139 (0.493)	0.027	0.778
Whole‐brain exponent				−1.807 (3.235)	−0.034	0.577
Neuroticism				7.084 (0.972)	**0.487**	**< 0.001**
Extraversion				−2.888 (0.921)	**−0.188**	**0.002**
Openness				0.866 (0.930)	0.051	0.354
Agreeableness				2.582 (1.246)	**0.119**	**0.040**
Conscientiousness				−5.846 (1.024)	**−0.348**	**< 0.001**
**B. Predicting Trait Depression (Whole‐brain Exponent)**						
**Step 1: Demographics**	0.002	0.002	0.120			0.948
**Step 2: + Exponent**	0.015	0.013	2.031			0.156
Sex				0.730 (1.500)	0.042	0.627
Age				0.275 (0.592)	0.067	0.643
Education				−0.449 (0.691)	−0.093	0.517
Whole‐brain exponent				6.089 (4.273)	0.123	0.156
**Step 3: + Big Five**	0.523	0.508	32.796***			**< 0.001**
Sex				−1.433 (1.095)	−0.082	0.193
Age				−0.249 (0.430)	−0.060	0.563
Education				0.070 (0.497)	.014	0.888
Whole‐brain exponent				−5.314 (3.257)	−0.107	0.105
Neuroticism				4.984 (0.979)	**0.368**	**< 0.001**
Extraversion				−5.644 (0.927)	**−0.395**	**< 0.001**
Openness				−0.246 (0.937)	−0.016	0.793
Agreeableness				0.298 (1.255)	0.015	0.813
Conscientiousness				−2.608 (1.031)	**−0.167**	**0.012**

*Note*: β = standardized coefficient. For Trait Anxiety (Panel A): Exponent β changed from 0.224 (*p* = 0.009) at Step 2 to −0.034 (*p* = 0.577) at Step 3, representing 84.8% attenuation. For Trait Depression (Panel B): Exponent was not significant at Step 2 (*p* = 0.156).

### 4.4 Mediation Analyses

Mediation results were presented in Table 5. The whole‐brain exponent showed a significant indirect association with trait anxiety through neuroticism in the simple mediation model, whereas the direct effect was no longer significant after neuroticism was included. In the parallel multiple mediation model, the total indirect effect remained significant. Neuroticism showed the largest indirect effect, followed by conscientiousness, whereas agreeableness showed a smaller indirect effect in the opposite direction.

**TABLE 5:** Mediation analyses: Whole‐brain aperiodic exponent → Psychological outcomes (*N* = 164).

Path / Effect	Effect	Boot SE	*t*	*p*	95% CI LL	95% CI UL	% of total
**A. Simple Mediation: Exponent → Neuroticism → Trait Anxiety**							
a (Exponent → Neuroticism)	1.351	0.298	4.531	**< 0.001**			
b (Neuroticism → Trait Anxiety)	10.047	0.903	11.125	**< 0.001**			
c (total effect)	11.917	4.521	2.636	**0.009**			
c′ (direct effect)	−1.660	3.608	−0.460	0.646			
**a × b (indirect effect)**	**13.576**	**3.230**			**7.472**	**20.244**	
**Standardized indirect**	**0.254**				**0.140**	**0.379**	
**B. Parallel Mediation: Exponent → Big Five → Trait Anxiety**							
Total effect (c)	11.917						
Direct effect (c′)	−1.807						
*Specific indirect effects*:							
**via Neuroticism**	**9.572**	**2.420**			**5.197**	**14.624**	**80.3%**
via Extraversion	1.319	0.894			−0.508	3.059	11.1%
via Openness	−0.512	0.727			−2.145	0.821	−4.3%
**via Agreeableness**	**−1.373**	**0.757**			**−3.065**	**−0.116**	**−11.5%**
**via Conscientiousness**	**4.717**	**1.903**			**1.384**	**8.800**	**39.6%**
**Total indirect**	**13.724**	**3.581**			**6.945**	**20.948**	
**C. Simple Mediation: Exponent → Neuroticism → Trait Depression**							
a (Exponent → Neuroticism)	1.351	0.298	4.531	**<.001**			
b (Neuroticism → Trait Depression)	8.556	0.914	9.357	**<.001**			
c (total effect)	6.089	4.271	1.426	.156			
c′ (direct effect)	−5.471	3.661	−1.494	.136			
**a × b (indirect effect)**	**11.561**	**2.884**			**6.299**	**17.765**	
**Standardized indirect**	**0.233**				**0.127**	**0.357**	
**D. Parallel Mediation: Exponent → Big Five → Trait Depression**							
Total effect (c)	6.089						
Direct effect (c′)	−5.314						
*Specific indirect effects*:							
**via Neuroticism**	**6.734**	**2.027**			**3.351**	**11.230**	**110.6%**
via Extraversion	2.578	1.710			−0.807	5.983	42.3%
via Openness	0.145	0.643			−1.207	1.492	2.4%
via Agreeableness	−0.158	0.709			−1.722	1.189	−2.6%
**via Conscientiousness**	**2.105**	**1.271**			**0.097**	**5.035**	**34.6%**
**Total indirect**	**11.403**	**3.295**			**5.013**	**18.042**	

*Note*: Covariates: sex, age, years of education. Bootstrap: 10,000 resamples, bias‐corrected and accelerated 95% CI. Bold rows indicate significant indirect effects (95% CI excludes zero). Panels A and C share the same *a* path (Exponent → Neuroticism). In Panel C, total effect *c* was not significant; indirect‐only mediation. Percentages exceeding 100% reflect opposing direct and indirect effects.

For trait depression, the whole‐brain exponent did not show a significant total effect, but significant indirect effects were observed through neuroticism in the simple mediation model and through neuroticism and conscientiousness in the parallel mediation model. Parallel analyses using whole‐brain offset yielded a similar pattern, with significant indirect effects for both trait anxiety and trait depression despite non‐significant direct effects.

Parallel mediation analyses were conducted for the whole‐brain offset to verify the robustness of the findings. For trait anxiety, the indirect effect through neuroticism was significant (ab = 6.270, 95% CI [2.045, 10.696]), while the direct effect was not (c′ = 1.413, *p* = 0.550). For trait depression, the total effect was not significant (c = 2.742, *p* = 0.345), but the indirect effect was significant (ab = 5.333, 95% CI [1.737, 9.131]) (Table S1). Some percentages of the total effect exceeded 100% because the direct and indirect effects were opposite in direction, indicating an inconsistent mediation or statistical suppression pattern. Because all variables were measured cross‐sectionally, these indirect effects do not establish temporal ordering or causal mediation.

## 5 Discussion

This study investigated, in an adult sample, how resting‐state EEG aperiodic parameters relate to both the Big Five personality traits and negative affective vulnerability, examining statistical indirect effects through personality traits. Overall, aperiodic neural activity was more consistently associated with personality traits than with negative affective vulnerability directly, and personality traits may statistically account for part of the association between aperiodic activity and affective vulnerability.

Among all psychological variables examined, neuroticism exhibited the most robust association with the aperiodic exponent, characterized by both the largest effect sizes and the most extensive spatial distribution. This finding may be interpreted through the perspective of cortical E/I balance. According to Gao et al. (2017), greater aperiodic exponent values are theorized to signify a shift toward inhibitory dominance within cortical E/I dynamics. This interpretation is supported by previous magnetic resonance spectroscopy (MRS) studies demonstrating positive associations between neuroticism and both glutamine levels and the glutamine‐to‐glutamate ratio in the left dorsolateral prefrontal cortex, suggesting dysregulated excitatory neurotransmitter dynamics in this personality trait (Hasler et al. 2019). Hettema and colleagues (2006) demonstrated that neuroticism was significantly associated with multiple polymorphic variants of *GAD1*, the gene encoding glutamate decarboxylase 1, a rate‐limiting enzyme in GABA synthesis. These findings furnish genetic evidence supporting an intrinsic coupling between neuroticism and GABAergic inhibitory neurotransmission. Converging evidence suggests that higher neuroticism may be characterized by elevated tonic excitatory activity during rest, with the observed steeper aperiodic exponent potentially representing a compensatory upregulation of cortical inhibition to stabilize E/I balance. Notably, Pacheco et al. were the first to systematically disentangle periodic and aperiodic EEG signals in personality research; their multivariate pattern analysis indicated that agreeableness was the sole trait consistently decodable from aperiodic exponent topography (2024). Moreover, Fruehlinger et al. (2024) failed to replicate any of Pacheco et al.’s principal findings in a larger independent sample using a comparable analytic pipeline. Collectively, these inconsistent findings suggest that the relationship between aperiodic neural activity and personality traits remains unstable, potentially reflecting sensitivity to methodological variations, sample characteristics, and cultural context. In this light, theory‐driven analytical approaches may prove more effective in establishing robust, systematic links between specific neural indices and psychological variables. However, this E/I should be regarded as a theoretical framework, because the aperiodic exponent is an indirect and model‐dependent proxy rather than a direct physiological measure of cortical excitation or inhibition.

In addition, the association between whole‐brain aperiodic exponent and trait anxiety was no longer statistically significant after the Big Five personality traits were included in the model. Neuroticism showed the largest indirect effect estimate, followed by conscientiousness, whereas agreeableness showed a smaller indirect effect in the opposite direction. These findings were statistically compatible with the proposed model in which personality traits account for part of the association between aperiodic activity and trait anxiety. This ordering was theory‐guided, given previous evidence identifying neuroticism as an important risk factor for anxiety and depressive disorders (Kotov et al. 2010; Ormel et al. 2013), but it should not be interpreted as an established temporal sequence. Alternative models in which personality traits precede aperiodic activity, affective vulnerability is positioned earlier in the sequence, or shared upstream factors contribute to all three domains may be equally plausible. Because all variables were measured cross‐sectionally, the present data cannot discriminate among these competing models or establish temporal or causal ordering.

Importantly, trait depression was not directly associated with any aperiodic parameter, whereas the indirect effect of the aperiodic exponent through neuroticism remained significant. This dissociation suggests that trait anxiety and the aperiodic exponent may be linked through a shared neurophysiological pathway involving neuroticism, while trait depression appears to engage alternative neural substrates. This interpretation aligns broadly with Clark and Watson's tripartite model, which posits that anxiety is characterized by heightened physiological arousal, whereas depression is distinguished by low positive affect (Kircanski et al. 2017; Khazanov and Ruscio 2016). The E/I balance dynamics reflected in aperiodic parameters may be more directly related to the physiological arousal in anxiety, whereas depression‐related reward dysfunction and motivational deficits may depend on other neural mechanisms. Consistent with this interpretation, Li et al. (2025) found reduced aperiodic offset in the parieto‐occipital region among patients with first‐episode, drug‐naïve major depressive disorder. These findings suggest that despite high clinical comorbidity, anxiety and depression may have partially dissociable aperiodic neural signatures. Future research should further clarify the specific contributions of aperiodic offset and periodic oscillatory features in depression. However, this apparent dissociation should be interpreted cautiously because the predominantly young, female, and largely university‐based sample showed restricted variability in trait depression scores, which may have attenuated the observed associations and reduced sensitivity to detect relationships with aperiodic parameters.

Furthermore, topographical analyses indicated that the association between neuroticism and the aperiodic exponent was widely distributed across the frontal, central, parietal, and temporal regions, implicating a diffuse, large‐scale network signature. In contrast, the association between trait anxiety and the aperiodic exponent was more concentrated at the whole‐brain level and within frontal regions. This topographic divergence implies that neuroticism may reflect a more global background feature of brain function, while trait anxiety may be more closely linked to neural activity patterns with a frontal predominance. This is consistent with the critical role of the frontal lobe, particularly the prefrontal cortex, in emotional evaluation, cognitive control, and emotion regulation (Ochsner and Gross 2005; Buhle et al. 2014). This finding suggests that anxiety‐related abnormalities in aperiodic activity may be more closely associated with prefrontal regulatory processes.

Habitual emotion regulation strategies may represent an additional psychological factor relevant to the observed associations among personality traits, resting‐state EEG activity, and negative affective vulnerability. Personality traits, particularly neuroticism, are closely related to the habitual use of strategies such as rumination, expressive suppression, and cognitive reappraisal (Barańczuk 2019). Previous resting‐state EEG studies have reported associations between emotion regulation strategies and frequency‐specific EEG complexity, spectral synchronization, and graph‐theoretical measures of large‐scale brain networks (Aydın and Akın 2022; Aydın 2023; Aydın 2022). Although these studies did not directly examine aperiodic parameters, they suggest that habitual emotion regulation may be related to the organization of resting‐state neural activity. Emotion regulation strategies could therefore plausibly mediate or moderate the observed associations involving personality traits and negative affective vulnerability. Because emotion regulation was not assessed in the present study, these possibilities could not be examined and should be investigated in future studies using instruments such as the Emotion Regulation Questionnaire or Cognitive Emotion Regulation Questionnaire.

## 6 Limitations

This study has several limitations. First, the cross‐sectional design precludes causal inference, and the proposed mediation pathway should therefore be interpreted as a pattern of associations rather than a definitive temporal sequence. Future longitudinal and experimental studies are needed to validate these pathways. Second, although the inclusion criteria allowed participants aged 18–55 years, the final sample was concentrated in young adulthood, with a mean age of approximately 20 years. The sample was also predominantly female and largely university‐based, which may limit the generalizability of the findings to broader adult and clinical populations. In addition, the restricted variability in trait depression scores may have attenuated the observed associations and contributed to the absence of significant direct relationships between trait depression and aperiodic parameters. Consequently, the null findings for trait depression should not be generalized to clinical populations, older adults, or populations with greater symptom variability. Third, the analyses were restricted to eyes‐closed resting‐state EEG, and aperiodic dynamics under task‐evoked conditions might show different patterns of association. Fourth, although sex was included as a covariate in the statistical analyses, menstrual cycle phase, circulating estradiol and progesterone levels, and the use of hormonal contraceptives were not assessed or controlled. Given evidence that reproductive hormonal fluctuations may modulate basal cortical activity, neuronal excitability, and resting‐state EEG properties (García‐Granados et al. 2026), this unmeasured hormonal variability may have influenced the aperiodic parameters and contributed to residual confounding. Future studies should assess menstrual cycle phase and hormonal contraceptive use and, where feasible, directly measure circulating hormone levels. Fifth, given their potential associations with personality traits, negative affective vulnerability, and resting‐state EEG dynamics, unmeasured individual differences in emotion regulation may have contributed to the observed associations or modified their strength. Finally, the interpretation of the aperiodic exponent in terms of cortical E/I balance remains debated and requires further validation using direct or multimodal physiological measures.

## 7 Conclusions

In conclusion, resting‐state EEG aperiodic parameters were systematically associated with personality and affective traits. The association between whole‐brain aperiodic exponent and trait anxiety was no longer statistically significant after the Big Five personality traits were included in the model, and significant indirect effects were observed, particularly through neuroticism. These findings suggest that personality traits may statistically account for part of the association between aperiodic activity and trait anxiety. Because the data were cross‐sectional, the temporal and causal direction of these associations cannot be determined. The findings highlight the potential utility of separating periodic and aperiodic EEG components when examining individual differences in personality and negative affective vulnerability. Given the narrow age distribution of the sample, the generalizability of these findings to middle‐aged and older adults remains to be established.

## Author Contributions

J. L. contributed to conceptualization, methodology, formal analysis, data curation, visualization, writing – original draft, and writing – review and editing. Z. D. contributed to investigation, data curation, EEG data preprocessing, and writing – review and editing. S. F. contributed to investigation, data collection, questionnaire administration, and writing. R. S. contributed to investigation, data collection, EEG data acquisition, and data curation. X. J. contributed to investigation, data collection, and data curation. H. X. contributed to investigation, EEG data acquisition, and data curation. Z. Z. contributed to investigation, questionnaire administration, and data curation. H. L. contributed to methodology, formal analysis, supervision, interpretation of findings, and writing – review and editing. H. J. contributed to conceptualization, supervision, project administration, resources, interpretation of findings, and writing – review and editing. H. L. and H. J. are co‐corresponding authors of this manuscript. All authors read and approved the final manuscript.

## Funding

This study was supported by the National Natural Science Foundation of China (82474429, 82305193), Capital Medical University Clinical‐Basic Cooperation Platform Cultivation Project (JLPYPT2025002), Beijing Traditional Chinese Medicine Inheritance “New 3+3” Program Demonstration Case Project (2023‐ZYSF‐19), and Capital Health Development Scientific Research Special Project (2026‐2‐2124). The funding sources had no role in the study design, data collection, analysis, interpretation of data, writing of the manuscript, or decision to submit the article for publication.

## Ethics Statement

The study was approved by the Ethics Committee of Beijing Anding Hospital, Capital Medical University (approval no. (2025) Research Ethics Approval No. 123). All participants provided written informed consent prior to their participation. The study was conducted in accordance with the Declaration of Helsinki.

## Conflicts of Interest

The authors declare no conflicts of interest.

## Supporting information




**Supplementary Table**: brb371836‐sup‐0001‐TableS1.docx

## Data Availability

The datasets generated and analyzed during the current study are not publicly available due to privacy and ethical restrictions but are available from the corresponding author on reasonable request and with appropriate ethical approval.
