# Supplementary material for: Resting‐State EEG Aperiodic Activity and Negative Affective Vulnerability: The Mediating Role of Big Five Personality Traits
Source: Brain Behav. 2026 Oct 21;16(10):e71836. doi: 10.1002/brb3.71836 (PMC13687659; doi:10.1002/brb3.71836)
Supplement: Supplementary file 1 [file BRB3-16-e71836-s001.docx]

**Table S1** Mediation Analyses: Whole-brain Aperiodic Offset → Psychological Outcomes (*N* = 164)

| Path / Effect | Effect | Boot SE | *t* | *p* | 95% CI LL | 95% CI UL | % of total |
| --- | --- | --- | --- | --- | --- | --- | --- |
| **A. Simple Mediation: Offset → Neuroticism → Trait Anxiety** | | | | | | | |
| a (Offset → Neuroticism) | 0.641 | 0.208 | 3.087 | **.002** |  |  |  |
| b (Neuroticism → Trait Anxiety) | 9.782 | 0.924 | 10.590 | **<.001** |  |  |  |
| c (total effect) | 7.682 | 3.055 | 2.515 | **.013** |  |  |  |
| c′ (direct effect) | 1.413 | 2.362 | 0.598 | .550 |  |  |  |
| **a × b (indirect effect)** | **6.270** | **2.206** |  |  | **2.045** | **10.696** |  |
| **Standardized indirect** | **0.160** |  |  |  | **0.052** | **0.273** |  |
| **B. Parallel Mediation: Offset → Big Five → Trait Anxiety** | | | | | | | |
| Total effect (c) | 7.682 |  |  |  |  |  |  |
| Direct effect (c′) | 1.174 |  |  |  |  |  |  |
| *Specific indirect effects:* | | | | | | | |
| **via Neuroticism** | **4.391** | **1.533** |  |  | **1.452** | **7.522** | **57.2%** |
| via Extraversion | 0.435 | 0.606 |  |  | −0.890 | 1.605 | 5.7% |
| via Openness | −0.131 | 0.269 |  |  | −0.776 | 0.355 | −1.7% |
| via Agreeableness | −0.435 | 0.453 |  |  | −1.507 | 0.305 | −5.7% |
| **via Conscientiousness** | **2.248** | **1.149** |  |  | **0.190** | **4.678** | **29.3%** |
| **Total indirect** | **6.509** | **2.343** |  |  | **1.778** | **11.045** |  |
| **C. Simple Mediation: Offset → Neuroticism → Trait Depression** | | | | | | | |
| a (Offset → Neuroticism) | 0.641 | 0.208 | 3.087 | **.002** |  |  |  |
| b (Neuroticism → Trait Depression) | 8.321 | 0.937 | 8.882 | **<.001** |  |  |  |
| c (total effect) | 2.742 | 2.892 | 0.948 | .345 |  |  |  |
| c′ (direct effect) | −2.591 | 2.411 | −1.074 | .281 |  |  |  |
| **a × b (indirect effect)** | **5.333** | **1.881** |  |  | **1.737** | **9.131** |  |
| **Standardized indirect** | **0.146** |  |  |  | **0.048** | **0.250** |  |
| **D. Parallel Mediation: Offset → Big Five → Trait Depression** | | | | | | | |
| Total effect (c) | 2.742 |  |  |  |  |  |  |
| Direct effect (c′) | −2.095 |  |  |  |  |  |  |
| *Specific indirect effects:* | | | | | | | |
| **via Neuroticism** | **3.066** | **1.223** |  |  | **0.975** | **5.734** | **111.8%** |
| via Extraversion | 0.835 | 1.090 |  |  | −1.521 | 2.850 | 30.4% |
| via Openness | 0.014 | 0.214 |  |  | −0.433 | 0.497 | 0.5% |
| via Agreeableness | −0.077 | 0.274 |  |  | −0.728 | 0.456 | −2.8% |
| via Conscientiousness | 0.999 | 0.656 |  |  | −0.036 | 2.462 | 36.4% |
| **Total indirect** | **4.837** | **2.137** |  |  | **0.501** | **8.925** |  |

*Note.* Covariates: sex, age, years of education. Bootstrap: 10,000 resamples, bias-corrected and accelerated 95% CI. Bold indirect effects indicate the 95% CI excludes zero. % of total = specific indirect effect / total effect × 100%. Panels A and C share the same *a* path (Offset → Neuroticism). In Panel C, total effect *c* was not significant; indirect-only mediation (Zhao et al., 2010).
